# Differential COVID-19 infection rates in children, adults, and elderly: Systematic review and meta-analysis of 38 pre-vaccination national seroprevalence studies

**DOI:** 10.7189/jogh.13.06004

**Published:** 2023-01-20

**Authors:** Cathrine Axfors, Angelo Maria Pezzullo, Despina G Contopoulos-Ioannidis, Alexandre Apostolatos, John PA Ioannidis

**Affiliations:** 1 Meta-Research Innovation Center at Stanford (METRICS), Stanford University, Stanford, California, USA; 2 Department for Women’s and Children’s Health, Uppsala University, Uppsala, Sweden; 3 Section of Hygiene, Department of Life Sciences and Public Health, Università Cattolica del Sacro Cuore, Rome, Italy; 4 Division of Infectious Diseases, Department of Pediatrics, Stanford University School of Medicine, Stanford, California, USA; 5 Faculty of Medicine, Université de Montréal, Montreal, Canada; 6 Departments of Medicine, of Epidemiology and Population Health, of Biomedical Data Science, and of Statistics, Stanford University, Stanford, California, USA

## Abstract

**Background:**

Debate exists about whether extra protection of elderly and other vulnerable individuals is feasible in COVID-19. We aimed to assess the relative infection rates in the elderly vs the non-elderly and, secondarily, in children vs adults.

**Methods:**

We performed a systematic review and meta-analysis of seroprevalence studies conducted in the pre-vaccination era. We identified representative national studies without high risk of bias through SeroTracker and PubMed searches (last updated May 17, 2022). We noted seroprevalence estimates for children, non-elderly adults, and elderly adults, using cut-offs of 20 and 60 years (or as close to these ages, if they were unavailable) and compared them between different age groups.

**Results:**

We included 38 national seroprevalence studies from 36 different countries comprising 826 963 participants. Twenty-six of these studies also included pediatric populations and twenty-five were from high-income countries. The median ratio of seroprevalence in elderly vs non-elderly adults (or non-elderly in general, if pediatric and adult population data were not offered separately) was 0.90-0.95 in different analyses, with large variability across studies. In five studies (all in high-income countries), we observed significant protection of the elderly with a ratio of <0.40, with a median of 0.83 in high-income countries and 1.02 elsewhere. The median ratio of seroprevalence in children vs adults was 0.89 and only one study showed a significant ratio of <0.40. The main limitation of our study is the inaccuracies and biases in seroprevalence studies.

**Conclusions:**

Precision shielding of elderly community-dwelling populations before the availability of vaccines was indicated in some high-income countries, but most countries failed to achieve any substantial focused protection.

**Registration:**

Open Science Framework (available at: https://osf.io/xvupr)

Coronavirus disease 2019 (COVID-19) is characterized by a steep age-gradient in risk of serious disease and death [1-3]. Death risk after infection increases approximately 3-fold per 10-year increments, thus differing more than a thousand-fold between pediatric and geriatric populations. The total fatalities footprint of a pandemic with such strong risk stratification is expected to depend on how effectively high-risk, vulnerable individuals are protected from infection [4]. This is particularly important for the pre-vaccination period, but remains relevant even as effective interventions such as vaccines are carried out.

The ability to more aggressively protect the elderly and other vulnerable individuals (ie, precision shield) has been contested [5,6]. For a widely circulating virus, it may be difficult to effectively shield only high-risk individuals. Nursing home residents, a particularly high-risk group of elderly people, were even disproportionately more frequently infected early in the pandemic [7-11]. Infections were massively spread in such facilities, as testified by high death tolls and high seroprevalence rates in their populations [9-13]. However, the question of age-stratified precision shielding remains open for community-dwelling populations. It is possible that infection rates vary in different age groups. Perhaps community-dwelling elderly might have been less mobile and less exposed than other adults. Conversely, children and adolescents may have had lower infection rates, given the widely implemented school closures.

Insights on the relative infection rates across age strata can be obtained from seroprevalence studies. Hundreds of such studies have been conducted to date [14]. However, such surveys are also susceptible to numerous biases [15]. To answer the question of whether age-specific precision shielding was achieved in the pre-vaccination period, we performed a systematic review and meta-analysis of national seroprevalence studies without high risk of bias. Through the meta-analysis, we aimed to estimate the relative infection rates in the elderly vs the non-elderly and, secondarily, in children vs adults.

## METHODS

We pre-registered this meta-analysis as part of a broader ongoing project on COVID-19 seroprevalence and infection fatality (protocol: https://osf.io/xvupr). Protocol amendments and clarifications are available in the Supplementary Table 1 in the **Online Supplementary Document**.

### Search strategy and eligibility criteria

We identified seroprevalence studies in the live systematic review SeroTracker [14-16]. We also performed PubMed searches using the string “seroprevalence AND (national OR stratified) AND COVID-19”. The initial search performed on February 8, 2022 was updated on May 17, 2022.

We included studies on SARS-CoV-2 seroprevalence had a national, representative sample, completed the sampling by February 28, 2021, included adults (with or without children and/or adolescents), and provided seroprevalence for non-elderly people (preferably for <70 years and/or <60 years, but any cut-off between 60 and 70 years was acceptable). We excluded studies focusing on patient cohorts, blood donors, workers, and insurance applicants and any other study where the examined population might have had lower or higher risk than the general population. In SeroTracker, only studies in the categories of “Household and community samples” and “Multiple general populations” without high risk of bias (reported by the SeroTracker team using the Joanna Briggs Institute (JBI) Critical Appraisal Tool for Prevalence Studies) were considered for further scrutiny. Similar criteria were applied to any additional PubMed-retrieved studies. Following previous work [17], for studies done in the USA, we retained only those that have adjusted the seroprevalence estimates for race/ethnicity [18].

For studies with several sampled (sub)regions of a country, we accepted those where the sampling locations might reasonably represent the entire country. Conversely, we excluded studies when only urban populations or when only rural populations were sampled, or when locations were selected only when hard hit or only when lightly hit. One reviewer selected the studies for the title/abstract review and two reviewers independently for full text eligibility.

We excluded seroprevalence studies where crude overall seroprevalence in the population was less than one-test specificity and/or the 95% confidence interval (CI) of the seroprevalence went to 0%, since the uncertainty on seroprevalence for them is very large.

To avoid any substantive impact of vaccination, we only considered seroprevalence studies where the sampling had been completed by the end of February 2021 and at least 90% of the samples had been collected before end of January 2021.

### Extracted information

Two authors independently performed the data extraction for eligible articles in duplicate, discussing any disagreements between themselves and consulting a third author (JPAI) if consensus was not achieved.

We extracted information on country, dates of sample collection, overall sample size (number tested) and sample size in pediatric, non-elderly adults, and elderly populations, and types of antibodies measured (immunoglobulin G (IgG), immunoglobulin G (IgM), immunoglobulin A (IgA)) from all eligible seroprevalence studies. We also extracted the estimated unadjusted seroprevalence (positive samples divided by all samples tested) and the most fully adjusted seroprevalence in children, non-elderly adults, and the elderly. We also noted the factors that the authors considered for adjustment in the most fully adjusted calculations. If multiple different time points when seroprevalence was assessed existed in a study, we selected the time point that gives the highest overall seroprevalence estimate; when there was a tie, we chose the earliest time point.

We defined groups of children (including adolescents), non-elderly adults, and elderly according to preferred age cut-offs of 20 years and 60 years; therefore, these groups ideally referred to 0-20 years, 21-60 years, and >60 years, respectively. For separating pediatric and non-elderly adult populations, we accepted cut-offs in the range 14-20, preferring the one available that was closest to 20. For separating non-elderly adults from elderly, we accepted cut-offs in the range of 54-70, preferring the one available that was closest to 60. Available seroprevalence data on more granular age strata were merged within the three main age groups.

### Data synthesis

For each eligible study, we calculated ratios of seroprevalence across children/adolescents, non-elderly adults, and elderly adults. For the main analysis, we focused on the ratio of seroprevalence in the elderly vs non-elderly (non-elderly adults or any non-elderly, if pediatric and adult population data were not offered separately). In the sensitivity analyses, we examined the ratios of seroprevalence in the elderly vs any non-elderly, and elderly vs strictly non-elderly adults. These ratios are “shielding ratios” [4] and allow for the evaluation of whether elderly individuals (a high-risk group) were more protected (and if so, by how much) and if there were consistent patterns across different countries. The observed ratios may thus provide estimates of the extent of precision shielding achieved in different countries [4] under the assumption that selection biases in sampling, test performance, and seroreversion are not substantially different in different age strata. We performed calculations using the crude numbers (tested positive/total tested) in each age stratum; when these were not available, we used the adjusted seroprevalence estimates and converted the adjusted proportion to an equivalent number of seropositives. When both crude numbers and adjusted estimates were available, we examined whether the latter changed the results.

In the secondary analysis, we examined the ratio of seroprevalence in children/adolescents vs non-elderly adults to evaluate whether there was preferential shielding of pediatric populations.

We had anticipated that substantial heterogeneity may exist across countries to preclude formal data synthesis by meta-analysis. Therefore, we expressed the results by using medians and by describing studies with extreme values. We also formally estimated the between-study heterogeneity of these ratios using the *I*^2^ statistic [19]. In the exploratory analyses, we evaluated whether results differed in high-income countries vs other countries (assuming that perhaps focused protection might be more feasible in the former). Analyses were conducted in STATA (StataCorp, USA).

## RESULTS

### Eligible studies

**Figure 1:**
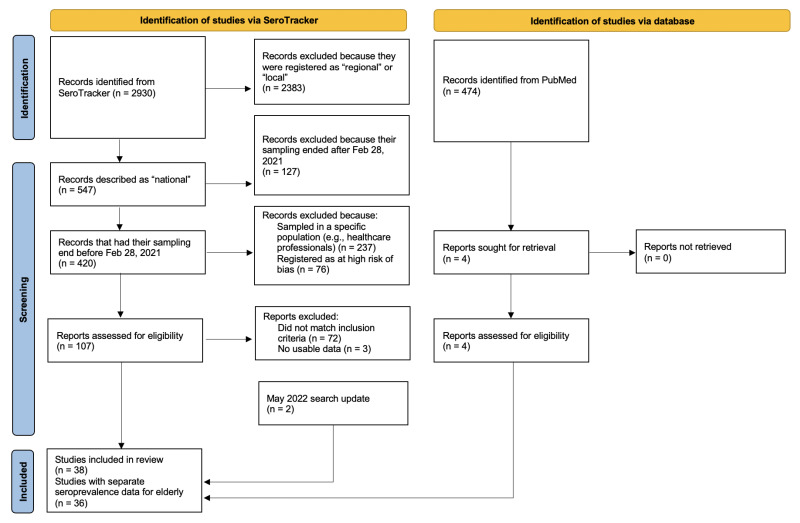
Flowchart for screening and selection of eligible studies. May 2022 search update: Among 3412 identified SeroTracker records, were manually assessed nine for eligibility (after applying relevant SeroTracker filters according to first search) and excluded seven. Details on the totally 79 reports excluded among 116 reports manually assessed for eligibility are available in Supplementary Table 2 in the **Online Supplementary Document**.

After in-depth screening (**Figure 1** and Supplementary Table 2 in the **Online Supplementary Document**), we included 38 eligible studies in the analyses: 36 had separate seroprevalence data on an elderly age stratum, while two (Afghanistan, Oman) could only separate pediatric vs adult population seroprevalence.

### Study characteristics

**Table 1** presents the 38 eligible studies. According to JBI risk of bias criteria, 18 studies [20-37] were deemed to be at low risk of bias and 19 [38-56] were deemed to be at moderate risk of bias. One study [57] was deemed at unclear risk of bias (no studies at high risk of bias were eligible upfront). The eligible studies came from 36 different countries (France and USA had two eligible studies each). More than half of the studies were performed in Europe (n = 20), 13 were performed in Asia, four in the Americas, and one in Africa. Twenty-five of the 38 studies came from high-income countries. Sample sizes varied substantially, but tended to be higher in high-income countries. Twenty-four studies had a total sample exceeding 5000, but this applied to only six out of 13 studies from non-high-income countries. Twenty-six studies provided separate data for a pediatric population with cut-off ages varying between 14 and 19 years, and 36/38 provided separate data for an elderly population with cut-offs varying between 54 and 70 years. Eleven studies assessed all antibodies, seven assessed IgG and IgM, and 20 assessed IgG only. Twenty studies performed all their sampling before or up to October 2020.

**Table 1:** Eligible studies – population and sampling details

Country	Children	Non-elderly adults	Elderly	Non-elderly	Age cut-off, pediatric	Age cut-off, elderly	Antibodies	Sampling time
Afghanistan	4346	5168		9514	17	NA	IgG, IgM	June 2020
Andorra	10 590	38 765	10 331	49 355	19	59	IgG, IgM	May 2020
Canada			1029	5789	NA	59	IgG only	May to September 2020
Czech Republic			1215	5665	NA	59	IgG only	December 2020 to January 2021
Denmark*	1126	13 500	3540	14 626	17	64	All	September to December 2020
England			21 953	77 955	NA	64	IgG only	June to July 2020
Faroe Islands	14 616	25 851	12 387	40 467	19	59	All	November 2020
France (Warszawski)	1438	47 555	14 531	48 993	17	64	IgG only	November 2020
France (Carrat)			41 933	40 193	NA	59	IgG only	May to September 2020
Germany			3819	11 302	NA	64	IgG only	October 2020 to February 2021
Hungary			2386	8088	NA	64	IgG only	May 2020
Iceland*			3400	27 176	NA	70	All	April to June 2020
India	2290	23 271	3037	25 561	17	60	IgG only	December 2020 to January 2021
Iran	2302	7596	1358	9898	17	59	IgG only	August to October 2020
Ireland	198	1224	311	1422	19	59	IgG only	June to July 2020
Israel	5864	32 809	15 687	38 673	19	59	IgG only	June to September 2020
Italy*	2788	21 434	12 176	24 222	17	59	IgG only	May to July 2020
Japan			2794	5156	NA	59	All	June 2020
Jersey			298	1077	NA	64	IgG, IgM	June 2020
Jordan	1486	3027	470	5513	19	59	IgG, IgM	December 2020 to January 2021
Lao PDR	233	1849	351	2082	18	60	IgG, IgM	August to September 2020
Lebanon	293	1449	200	1742	19	59	IgG only	December 2020 to January 2021
Lithuania			2218	868	NA	64	IgG, IgM	October 2020
Maldives	410	1396	83	1806	17	59	IgG only	October to November 2020
Mexico*	1891	5785	1787	7676	19	59	All	August to November 2020
Mongolia	1898	2784	317	4682	19	59	IgG only	October to December 2020
Nepal	455	2275	310	2730	14	64	All	October 2020
Netherlands	1128	3472	2213	4600	19	59	IgG only	June to August 2020
Norway	868	21 396	5436	22 264	19	66	IgG only	November 2020 to February 2021
Oman	57	4007		4064	14	NA	IgG only	November 2020
Pakistan	1995	2030	975	4022	19	59	IgG, IgM	October to November 2020
Portugal	2108	6495	4795	8603	17	54	All	September to October 2020
Russia	9705	44 921	19 432	54 626	17	59	IgG only	June to July 2020
Senegal	462	867	117	1329	15	60	All	October to November 2020
Slovenia*	174	673	364	847	20	60	All	October to November 2020
Spain	8636	27 453	15 320	36 089	19	59	IgG only	November 2020
USA (Sullivan)		3481	1173	3481	NA	64	All	August to December 2020
USA (Kalish)		6785	1273	6785	NA	69	All	April to August 2020

NA – not available, IgG – immunoglobin G, IgM – immunoglobin M

*Denmark – numbers are approximated based on number of invited persons and proportion participating. Iceland – numbers are approximated based on estimated age-stratified national seroprevalence, number of people tested and the population pyramid. Italy – numbers are approximated based on the proportion positive and the 95% CI in each age group. Mexico – numbers are approximated from adjusted seroprevalence. Slovenia – October 3 estimates used according to predefined eligibility criteria.

### Seroprevalence in the different age groups

**Table 2** shows the seroprevalence estimates for the pre-specified groups of children, non-elderly adults, non-elderly, and elderly. There was a wide range of values from 0% in Faroe Islands to over 40% in the Czech Republic. Whenever available, adjusted seroprevalence estimates tended to be similar to unadjusted estimates, with few exceptions (**Table 2**). Parameters used for adjustments are shown in Supplementary Table 3 in the **Online Supplementary Document**.

**Table 2:** Seroprevalence estimates (%) for age groups – unadjusted (and adjusted in parenthesis)

Country	Seroprevalence in children	Seroprevalence in non-elderly adults	Seroprevalence in non-elderly	Seroprevalence in elderly
Afghanistan	25.3	35.2	30.7	
Andorra	12.6	11.0	11.3	13.1
Canada			2.1	0.7
Czech Republic			43.7	41.6
Denmark	6.5 (6.5)	4.2 (4.3)	4.4 (4.5)	2.3 (2.3)
England			6.1 (6.8)	3.6 (3.2)
Faroe Islands	0.0	0.0	0.0	0.0
France (Warszawski)	8.9 (9.8)	6.7 (6.4)	6.8 (6.5)	4.2 (4.2)
France (Carrat)			6.8	2.3
Germany			1.3 (1.9)	1.1 (0.6)
Hungary			0.6 (0.7)	0.8 (0.8)
Iceland			1.0	0.5
India	27.7 (27.0)	25.6 (24.0)	25.7 (24.5)	28.2 (26.0)
Iran	(11.5)	(12.6)	(12.4)	(19.4)
Ireland	1.5 (1.4)	2.0 (1.7)	1.9 (1.7)	1.9 (1.7)
Israel	(7.2)	(4.0)	(4.5)	(2.2)
Italy	2.2 (2.2)	2.5 (2.5)	2.5 (2.5)	2.6 (2.6)
Japan			0.1	0.1
Jersey			3.8	5.4
Jordan	36.2	33.8	34.6	34.5
Lao PDR	3.9 (4.2)	4.9 (5.1)	4.8 (5.0)	8.8 (9.3)
Lebanon	15.4 (17.8)	15.9 (18.3)	15.8 (18.2)	18.5 (21.4)
Lithuania			1.4	2.1
Maldives	4.9	15.2	12.8	31.3
Mexico	22.5 (22.5)	27.8 (27.9)	26.5 (25.7)	18.6 (18.6)
Mongolia	1.1 (0.8)	1.7 (1.3)	1.5 (1.1)	1.3 (1.2)
Nepal	(8.8)	(15.7)	(14.1)	(10.7)
Netherlands	2.4 (3.7)	5.2 (4.9)	4.5 (4.2)	5.0 (4.9)
Norway	1.8 (1.9)	0.9 (0.8)	0.9 (0.9)	0.6 (0.5)
Oman	(23.5)	(21.5)	(21.5)	
Pakistan	4.2	8.9	6.6	8.7
Portugal	(2.4)	(2.3)	(2.3)	(1.9)
Russia*	21.6	15.6	16.6	17.4
Senegal	19.3 (19.3)	31.7 (32.1)	27.4 (27.4)	24.8 (25.1)
Slovenia*	4.9	5.6	5.5	2.1
Spain	7.8 (7.6)	10.4 (10.5)	9.8 (9.3)	10.4 (10.3)
USA (Sullivan)		5.6 (5.5)	5.6 (5.5)	2.9 (1.9)
USA (Kalish)		3.8 (4.1)	3.8 (4.1)	3.6 (3.5)

*Russia – median percentage across tested regions. Slovenia – October 3 estimates used according to predefined eligibility criteria. Inverse-variance fixed effects meta-analysis used to combine age sub-strata.

### Ratio of seroprevalence in different age groups

**Figure 2:**
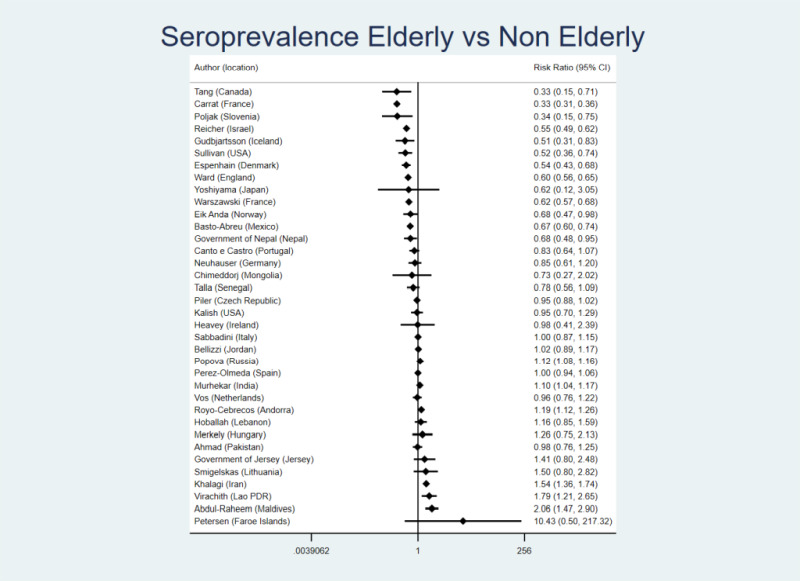
Seroprevalence ratio for the elderly vs non-elderly (non-elderly adults or non-elderly in general, if pediatric and adult population data were not offered separately). The definitions of age groups are detailed in the “Methods” section. We estimated the presented 95% CIs using crude counts in a two by two table for each study ((number elderly positive/number elderly tested)/(number non-elderly positive/number non-elderly tested)). When only adjusted seroprevalence estimates were available without crude data, we converted them to equivalent of number positive (number positive = adjusted seroprevalence × number tested in the specific age group).

**Figure 2** shows the ratio of seroprevalence in the elderly vs the seroprevalence in non-elderly (non-elderly adults or non-elderly in general, if pediatric and adult population data were not offered separately). We found large between-study variability, with *I*^2^ = 98%.

The median ratio was 0.95 (0.90 if adjusted seroprevalence estimates were given priority in the calculations), suggesting a slightly lower seroprevalence in elderly populations; 23/36 studies had point estimates in this direction. For the two countries with two studies each, the point estimates were in the same direction, but the magnitude of the estimated protection of the elderly varied. Twelve studies with suggested protection of elderly and six studies with suggested inverse protection (higher seroprevalence in the elderly) had 95% CIs excluding a ratio of 1.00. Canada, Slovenia, one of the two studies in France and (in adjusted analyses only) Germany, and one of the USA studies suggested protection over 2.5-fold (ratio <0.40) with 95% CIs excluding 1.00. We did not observe inverse protection of such magnitude (ratio >2.5) with 95% CIs excluding 1.00 in any study.

The sensitivity analyses gave similar results: the median ratio of seroprevalence in the elderly vs any non-elderly was 0.95, with 20/36 studies offering point estimates in the direction of some protection of the elderly; the median ratio of seroprevalence in the elderly vs strictly non-elderly adults was 0.98 with 14/24 studies offering point estimates in the direction of some protection in the elderly.

**Figure 3:**
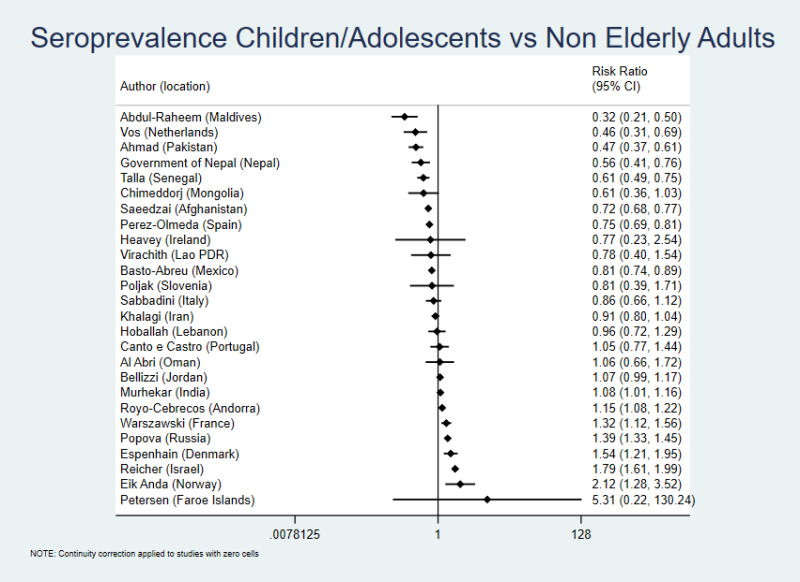
Seroprevalence ratio for pediatric populations vs non-elderly adults. The definitions of age groups are detailed in the “Methods” section. We estimated the presented 95% CIs using crude counts in a two by two table for each study ((number of pediatric population positive/number of pediatric population tested)/(number of non-elderly adults positive/number of non-elderly adults tested)). When only adjusted seroprevalence estimates were available without crude data, these were converted to equivalent of number positive (number positive = adjusted seroprevalence × number tested in the specific age group).

We also observed large between-study heterogeneity (*I*^2^ = 96%) in the comparison of pediatric populations vs non-elderly adults (**Figure 3**). The median ratio of seroprevalence was 0.89 and 15/26 studies presented point estimates in the direction of greater protection of children/adolescents than non-elderly adults. Fifteen studies had 95% CIs excluding a ratio of 1.00 (with lower seroprevalence in the pediatric populations in eight and higher in seven). Only one study (Maldives) showed a ratio of <0.40 with 95% CIs excluding 1.00 and none had a ratio >2.5 with 95% CIs excluding 1.00.

### High income vs non-high-income countries

For the main analysis of elderly vs non-elderly (the latter group comprising of non-elderly adults or non-elderly in general, if pediatric and adult population data were not offered separately), the median ratio was 0.85 in 25 studies done in high-income countries (0.83 if priority were given to adjusted estimates) and 1.02 in 11 studies done in non-high-income countries. All five statistically significant estimates of >2.5-fold protection of the elderly were in high-income countries. For a more modest protection threshold, all nine estimates of >1.5-fold protection of the elderly (ratio <0.67) with 95% CIs excluding 1.00 were in high income countries, while both estimates of >1.5-fold inverse protection (higher seroprevalence in the elderly) with 95% CIs excluding 1.00 were in non-high-income countries.

## DISCUSSION

Our analysis of data from 38 national seroprevalence studies for COVID-19 showed that, before the advent of massive vaccination, there was large heterogeneity across countries on the extent to which elderly people in the community were protected or not from infection compared with younger populations. On average, there was very little extra shielding of the elderly. However, several countries apparently did achieve substantial precision shielding of this vulnerable group. Conversely, in a few countries, the elderly were apparently infected slightly more frequently than non-elderly adults. Conclusive evidence for substantial preferential protection of the elderly in the community was seen only in some high-income countries. In non-high-income countries, the average ratio of seroprevalence between age groups suggested no preferential protection by age. There was also little difference in seroprevalence in children vs non-elderly adults overall, but the pattern differed across countries. On average, children were slightly less frequently infected than non-elderly adults.

These data suggest that precision shielding of vulnerable elderly populations is feasible, but strong shielding of the community-dwelling elderly populations was uncommon during the COVID-19 pandemic. It is unclear if this failure reflects practical difficulties of achieving major precision shielding, especially in disadvantaged settings [58,59], or the fact that pandemic response policies may not have focused much on this aspect, instead aiming for more horizontal measures. Country-level responses may have differed in this regard. Even within countries, heterogeneity may have existed across states and local communities on the timing and duration of specific measures that may protect some parts of the population more than others. For example, some measures may be more stringent on reducing the exposure of the elderly population (eg, avoidance of visits), while others (eg, school closures) might attempt to reduce exposure among children. While some may argue that the most draconian lockdown should be associated with equal protection of all age groups, this may not be so in practice, as (for example) essential workers who continue to be substantially exposed are non-elderly adults.

Given that a large share of COVID-19 deaths among community-dwelling people happen in the elderly, protection shielding exceeding 2.5-fold, as documented for five countries in our analysis, reflects roughly halving the total COVID-19 deaths among community-dwelling populations, showing that the benefit can be very significant. Unfortunately, however, the countries that apparently did achieve some substantial shielding of their community-dwelling elderly, failed in protecting resident of long-term care facilities [7-13], where infection fatality rates can be much higher (even 10-fold higher) than in community-dwelling elderly [17]. This resulted in numerous deaths of elderly residents in countries like USA, Canada, France, Germany, and Slovenia. Seroprevalence studies have documented extremely high rates of infection in nursing homes, much higher than in the community, in diverse countries, especially during 2020 [9-13]. Nursing home residents and other institutionalized people are routinely excluded from sampling in national seroprevalence studies and are, in any case, a small portion of the total population of any country.

Some limitations of our work should be discussed. First, we tried to select studies with maximal representativeness, but some samples may not have ended being fully representative, as some subpopulations are more difficult to recruit that may have different rates of infection than those recruited (e.g. the homeless and other marginalized groups). Second, both overall seropositivity and in specific age groups may vary temporally; for example, in some settings, an age group may have been protected or over-exposed compare to others at some point, depending on the measures taken. Third, screening schemes and approaches differed across countries, possibly creating heterogeneity in the results. Fourth, we focused on studies that were rated as having low or moderate risk of bias according to SeroTracker, but this does not offer absolute protection from many biases that may have affected the results. Fifth, given the unavoidable between-country heterogeneity, conclusions from the results of the meta-analysis need to be drawn carefully.

Additionally, the probability of seroconversion after infection and the rapidity of seroreversion may vary depending on age [60,61]. Elderly people may have initially stronger immune responses linked to more severe disease or weaker responses due to immune system senescence. If anything, old age and more symptomatic disease tend to be associated with longer persistence of antibodies [60]. If so, the precision shielding of elderly may have been slightly larger than what we calculated. Different antibody assays may also exhibit different rates of seroreversion and this may also vary per age group. However, given that most studies evaluated here were done early in the pandemic, seroreversion was probably not large.

For some studies, seroprevalence rates were very low and 95% CIs very wide. Depending on what adjustments are made, seroprevalence ratios might also differ in such cases, although in most studies we observed similar results for adjusted and unadjusted calculations.

The counterfactual seroprevalence ratios in the absence of any restrictive measures are unknown. Evidence from influenza seroprevalence assessments suggests that often children and/or young adults may be infected more frequently than elderly individuals, perhaps due to greater mobility and exposures, but this is not absolute and may vary per year and location [62-65]. Extrapolations to SARS-CoV-2 are tenuous.

Finally, focused protection may have varied in subsequent phases of the pandemic, with different infection rate ratios across age groups. Different waves due to different SARS-CoV-2 variants may have exhibited different age patterns and their underlying age-related variation in susceptibility and/or infectiousness is not well understood. Vaccine availability in 2021 was typically prioritized for the elderly leading to shifts in the age distribution of COVID-19 impact [66]. Vaccination also allowed more mobility and higher population exposure. After the Delta and Omicron waves, most people were infected at least once in most countries [67]. Even if precision shielding of the elderly can be achieved (as our data suggest), it is unknown whether it can be maintained effectively for pandemic-long circles lasting two or more years. Moreover, adverse consequences of trying to diminish exposures of vulnerable elderly may be substantial for their social well-being and their mental health [68,69]. Adverse consequences are likely for all age groups, including for children after school closures [70].

## CONCLUSIONS

Despite these limitations, we can conclude that precision shielding was feasible in several high-income countries in the first year of the pandemic, but most countries had no major differences in infection rates across age groups. Precision shielding remains an attractive concept given the extreme variability of fatality risk of SARS-CoV-2 infection across age groups [17,71], but when it is not achieved (or worse, when vulnerable individuals, such as elderly and nursing home residents are even more frequently infected than the non-vulnerable) the death toll and excess deaths become high [72]. These observations may be useful for future pandemic preparedness, especially for pathogens exhibiting large fatality rate variability across different population groups.

## Additional material


Online Supplementary Document
