# Supplementary material for: Differential COVID-19 infection rates in children, adults, and elderly: Systematic review and meta-analysis of 38 pre-vaccination national seroprevalence studies
Source: J Glob Health. 2023 Jan 20;13:06004. doi: 10.7189/jogh.13.06004 (PMC9850866; doi:10.7189/jogh.13.06004)
Supplement: Online Supplementary Document [file jogh-13-06004-s001.pdf]

ONLINE SUPPLEMENTARY DOCUMENT

Title: Differential COVID-19 infection rates in children, adults, and elderly: systematic review and meta-analysis of 38 pre-vaccination national seroprevalence studies

Authors: Cathrine Axfors, Angelo Maria Pezzullo, Despina G Contopoulos-Ioannidis, Alexandre Apostolatos, John PA Ioannidis

3 supplementary tables and supplementary references and PRISMA 2020 checklists.

Supplementary Table 1: Amendments/clarifications to protocol.

Manuscript section	Amendment/Clarification	Rationale
Eligibility for this subproject	For separating pediatric and non-elderly adult populations, we accepted cut-offs in the range 14-20, preferring the one available that was closest to 20. For separating non-elderly adults from elderly, we accepted cut-offs in the range of 54-70, preferring the one available that was closest to 60.	Clarification/specification needed for lower acceptable cut-off of pediatric group; for elderly cut-off accepted also one study (Portugal) with cut-off at 54 years (its exclusion gives similar results).
Analysis	The main analysis focused on the ratio of seroprevalence in the elderly versus non-elderly (non-elderly adults or non-elderly in general, if pediatric and adult population data were not offered separately). In sensitivity analyses, we examined the ratios of seroprevalence in the elderly versus any non-elderly, and elderly versus strictly non-elderly adults.	Clarification/specification needed
Analysis	Calculations were performed using the crude numbers (tested positive/tested) in each age stratum; when these were not available, we used the adjusted seroprevalence estimates and converted the adjusted proportion to an equivalent number of seropositives. When both crude numbers and adjusted estimates were available, we examined whether the latter changed results.	Clarification/specification needed; analyses were run with both crude and adjusted results and results are similar (as shown in the paper)
Analysis	The presented 95% confidence intervals for seroprevalence ratios are estimated using crude counts in a 2 by 2 table for each study. When only adjusted seroprevalence estimates were available without crude data, these were converted to equivalent of number positive (number positive = adjusted seroprevalence x number tested in the specific age group).	Clarification/specification needed

Supplementary Table 2. Details of excluded studies among reports manually assessed for eligibility (first search: 107 assessed, 72 excluded; search update: 9 assessed, 7 excluded).

URL/DOI	Country	Reason for exclusion
First search		
https://dx.doi.org/10.1038/s41598-021-92775-y	Brazil	Several sampled (sub)regions not representative of country
https://www.ncbi.nlm.nih.gov/pmc/articles/PMC8225319/	Brazil	Several sampled (sub)regions not representative of country
https://papers.ssrn.com/sol3/papers.cfm?abstract_id=3762489	Cabo Verde	Seroprevalence < 1 - test specificity
https://papers.ssrn.com/sol3/papers.cfm?abstract_id=3752659	Canada	Duplicate
https://papers.ssrn.com/sol3/papers.cfm?abstract_id=3933650	Chile	Several sampled (sub)regions not representative of country
https://dx.doi.org/10.1016/j.lanwpc.2021.100094	China	Several sampled (sub)regions not representative of country
https://dx.doi.org/10.1007/s10654-021-00796-8	Denmark	Duplicate
https://dx.doi.org/10.1007/s10654-021-00796-8	Denmark	Duplicate
https://dx.doi.org/10.1007/s10654-021-00796-8	Denmark	Duplicate
https://doi.org/10.1101/2021.05.07.21256725	Denmark	Not general population sample
https://www.medrxiv.org/content/10.1101/2021.08.10.21261777v1	Denmark	Seroprevalence < 1 - test specificity
https://dx.doi.org/10.1371/journal.ppat.1009413	Egypt	Several sampled (sub)regions not representative of country
https://ut.ee/et/sisu/koroonaviiruse-levimuse-seireuuringu-antikehade-analuusi-tulemused	Estonia	News article
http://dx.doi.org/10.1016/j.ijid.2021.08.028	Ethiopia	Several sampled (sub)regions not representative of country
https://dx.doi.org/10.1186/s12879-021-06973-0	France	Duplicate of included study
https://www.medrxiv.org/content/10.1101/2021.10.25.21265456v1.full-text	France	Duplicate (preprint of full paper)
https://drees.solidarites-sante.gouv.fr/sites/default/files/2021-01/er1167-en.pdf	France	Duplicate (preprint of full paper)
https://dx.doi.org/10.1016/j.idnow.2020.12.007	France	Several sampled (sub)regions not representative of country
https://papers.ssrn.com/sol3/papers.cfm?abstract_id=3834300	Germany	Several sampled (sub)regions not representative of country
https://www.ifo.de/en/publikationen/2020/monograph-authorship/die-deutschen-und-corona	Germany	Not general population sample

https://www.medrxiv.org/content/10.1101/2021.06.21.21259001v1.full-text	Germany	Not general population sample
https://dx.doi.org/10.3390/vaccines9050504	Greece	Not general population sample: Leftover samples
https://revistas.ucr.ac.cr/index.php/psm/article/view/43261/46175	Honduras	Municipalities with no reported cases
10.1101/2021.03.19.21253429	India	Several sampled (sub)regions not representative of country
https://dx.doi.org/10.1016/S2214-109X(20)30544-1	India	Duplicate
http://dx.doi.org/10.4103/ijmr.IJMR_3290_20	India	Duplicate of included study
https://www.bbc.com/news/world-asia-india-55945382	India	News article
https://www.medrxiv.org/content/10.1101/2021.02.05.21251118v2	India	Several sampled (sub)regions not representative of country
http://dx.doi.org/10.1016/S1473-3099%2820%2930858-6	Iran	Several sampled (sub)regions not representative of country
http://dx.doi.org/10.1016/S1473-3099%2820%2930858-6	Iran	Several sampled (sub)regions not representative of country
https://www.google.com/url?rct=j&sa=t&url=https://www.timesofisrael.com/initial-antibody-tests-indicate-200000-israelis-have-had-covid-19-report/&ct=ga&cd=CAAYBjIaOTBkZWE5ODk5NGU5MTg1OTpjb206ZW46VVM&usg=AFQjCNHYeXqD2hIEiNnZoH5qLiVlZKxQjg	Israel	News article
https://dx.doi.org/10.1128/Spectrum.00870-21	Israel	Seroprevalence < 1 - test specificity
https://www.istat.it/it/files//2020/08/ReportPrimiRisultatiIndagineSiero.pdf	Italy	Duplicate
http://dx.doi.org/10.1016/j.onehlt.2021.100292	Jordan	Duplicate
http://dx.doi.org/10.1016/j.onehlt.2021.100292	Jordan	Duplicate
https://dx.doi.org/10.3390/jcm9123989	Liechtenstein	Not general population sample
https://www.medrxiv.org/content/10.1101/2020.05.11.20092916v1	Luxembourg	Seroprevalence < 1 - test specificity
https://www.gob.mx/salud/prensa/255-secretaria-de-salud-presenta-resultados-preliminares-de-la-encuesta-nacional-de-salud-y-nutricion-covid-19?idiom=es	Mexico	News article
https://saludpublica.mx/index.php/spm/article/view/12847	Mexico	Not general population sample
https://saludpublica.mx/index.php/spm/article/view/12847	Mexico	Duplicate

https://dx.doi.org/10.1136/jech-2020-215678	Netherlands	Duplicate of included study
https://dx.doi.org/10.1016/j.ijid.2021.09.062	Oman	Duplicate
https://dx.doi.org/10.1016/j.ijid.2021.09.062	Oman	Duplicate
https://dx.doi.org/10.1016/j.ijid.2021.09.062	Oman	Duplicate
https://dx.doi.org/10.1007/s15010-021-01629-2	Pakistan	Several sampled (sub)regions not representative of country
https://world.kbs.co.kr/service/news_view.htm?lang=e&Seq_Code=157801	Republic of Korea	News article
https://www.google.com/url?rct=j&sa=t&url=http://www.koreaherald.com/view.php%3Fud%3D20200709000787&ct=ga&cd=CAAYEDIaOTBkZWE5ODk5NGU5MTg1OTpjb206ZW46VVM&usg=AFQjCNG8AlCeBcx2rR4FfgC726ll5TtgUw	Republic of Korea	News article
https://www.themoscowtimes.com/2020/06/10/1-in-7-russians-have-coronavirus-immunity-official-a70540 ; https://www.interfax.ru/russia/712617	Russia	News article
https://www.medrxiv.org/content/10.1101/2021.01.28.21250598v1	Saudi Arabia	Not general population sample
https://dx.doi.org/10.1016/j.cmi.2021.03.009	Slovenia	Duplicate
https://www.nicd.ac.za/wp-content/uploads/2021/03/COVID-19-Special-Public-Health-Surveillance-Bulletin-9-12-March-2021_.pdf	South Africa	Several sampled (sub)regions not representative of country
https://www.medrxiv.org/content/10.1101/2021.02.26.21252512v1	United Kingdom of Great Britain and Northern Ireland	Duplicate
https://www.medrxiv.org/content/10.1101/2021.02.26.21252512v1	United Kingdom of Great Britain and Northern Ireland	<90% by Jan 31, 2021
https://www.ukbiobank.ac.uk/media/x0nd5sul/ukb_serologystudy_report_revised_6months_jan21.pdf	United Kingdom of Great Britain and Northern Ireland	Not general population sample: Biobank
https://www.gov.uk/government/publications/national-covid-19-surveillance-reports	United Kingdom of Great Britain and Northern Ireland	Not general population sample
https://www.gov.uk/government/publications/national-covid-19-surveillance-reports	United Kingdom of Great Britain and Northern Ireland	Not general population sample
https://www.medrxiv.org/content/10.1101/2020.10.26.20219725v1.full.pdf	United Kingdom of Great Britain and Northern Ireland	Duplicate (preprint of full paper)

https://www.medrxiv.org/content/10.1101/2020.10.26.20219725v1.full.pdf	United Kingdom of Great Britain and Northern Ireland	Duplicate (preprint of full paper)
https://papers.ssrn.com/sol3/papers.cfm?abstract_id=3764198	United Kingdom of Great Britain and Northern Ireland	Not general population sample
https://papers.ssrn.com/sol3/papers.cfm?abstract_id=3764198	United Kingdom of Great Britain and Northern Ireland	Duplicate
https://papers.ssrn.com/sol3/papers.cfm?abstract_id=3764198	United Kingdom of Great Britain and Northern Ireland	Duplicate
https://www.gov.uk/government/publications/national-covid-19-surveillance-reports	United Kingdom of Great Britain and Northern Ireland	Seroprevalence < 1 - test specificity
https://dx.doi.org/10.1126/scitranslmed.abh3826	United States of America	Duplicate
https://jamanetwork.com/journals/jamanetworkopen/fullarticle/2777502?utm_source=For_The_Media&utm_medium=referral&utm_campaign=ftm_links&utm_term=031621	United States of America	Not general population sample: Life insurance
https://www.croiconference.org/abstract/us-population-based-survey-of-vaccine-willingness-and-sars-cov-2-antibody-prevalence/	United States of America	Meeting abstract
https://dx.doi.org/10.1136/bmjopen-2021-048778	United States of America	Not general population sample
https://pubmed.ncbi.nlm.nih.gov/33532807/	United States of America	Duplicate (report of full paper)
https://dx.doi.org/10.1016/j.ejim.2021.01.029	Vatican City	Not general population sample
http://dx.doi.org/10.3390/ijerph18126353	Viet Nam	Not general population sample: High-risk communities
http://dx.doi.org/10.3390/ijerph18126353	Viet Nam	Duplicate
http://dx.doi.org/10.3390/ijerph18126353	Viet Nam	Duplicate
https://www.thelancet.com/journals/langlo/article/PIIS2214-109X(21)00053-X/fulltext	Zambia	Several sampled (sub)regions not representative of country
Search update		
https://dx.doi.org/10.1001/jamanetworkopen.2021.46798	Canada	Duplicate of included study
https://dx.doi.org/10.1186/s12879-022-07045-7	Chile	Several sampled (sub)regions not representative of country
https://doi.org/10.1038/s43856-022-00080-0	Czechia	Overlapping with included study
https://dx.doi.org/10.1038/s41467-022-28232-9	Mexico	Duplicate of included study
https://dx.doi.org/10.2807/1560-7917.ES.2022.27.13.2100376	Norway	Duplicate of included study
https://dx.doi.org/10.1016/j.jcv.2022.105130	Spain	Duplicate of included study

https://dx.doi.org/10.1186/s12916-022-02286-4	United Kingdom of Great Britain and Northern Ireland	Not general population sample

Supplementary Table 3: Adjustments used in the adjusted seroprevalence estimates

Country	Adjustments made
Afghanistan	NA
Andorra	NA
Canada	NA
Czech Republic	NA
Denmark	Test sensitivity and specificity using the Rogan-Gladen estimator
England	Test performance, and weighted to account for sample design and for variation in response rate (age, sex, ethnicity, region and deprivation) to be representative of the England population over 18 years
Faroe Islands	NA
France (Warszewski)	Sample design, non-response, census calibration
France (Carrat)	NA
Germany	Non-response, test performance and seroreversion
Hungary	Design weighted. Response sample calibrated to known population counts by region, sex, and age categories.
Iceland	NA
India	Cluster sampling, sensitivity, specificity
Iran	Measurement error of the laboratory test, non-response bias and sampling design.
Ireland	Weighted to adjust for varying response rates in age-sex strata
Israel	Age, sex, time period, RT-PCR status, municipal strata, sampling
Italy	Non-response, region, age, sex, working status, province
Japan	NA
Jersey	NA
Jordan	NA
Lao PDR	Weighted for complex survey sample design, age and sex
Lebanon	Sex, age and area of residence, test performance

Lithuania	NA
Maldives	NA
Mexico	Test performance, and used sampling weights to adjust for selection probabilities and non-response rates (with post-stratification on region, sex and age group)
Mongolia	Test sensitivity and weighted prevalence rates according to age, sex, and provincial or district population size
Nepal	Survey design weights, age
Netherlands	Adjusted for survey design, weighted to match the distribution of the general Dutch population (based on sex, age, ethnic background, and degree of urbanization) and controlled for test characteristics
Norway	Rake weighting for population estimates of seroprevalence by age, sex, place of birth and county based on individual-level data for the invited sample (participants and non-responders) together with the corresponding distributions from the source population, provided by the Norwegian Population Register. Applied propensity scores for nonresponse adjustment and jackknife replicate weights for the raking procedure. Estimates subsequently corrected for test performance
Oman	Age group, sex, nationality
Pakistan	NA
Portugal	Adjusted for test performance, used sample weights and post-stratified by sex to adjust the seroprevalence extrapolating from the strata to the whole population
Russia	NA
Senegal	Weighted according to the age and sex distribution of the general population and adjusted test performance
Slovenia	NA
Spain	Characteristics of the random subsample of the fourth round
USA (Sullivan)	Test performance, design weights

USA (Kalish)	Age, region, sex, urban/rural, race, Hispanic, BRFSS survey response, sensitivity, specificity

NA, not available

Supplementary References

1. Saeedzai SA, Osmani A, Noormal B. Prevalence of COVID-19 and its related deaths in Afghanistan: a Nationwide, Population-Based Seroepidemiological Study. Islamic Republic of Afghanistan, Ministry of Public Health, Kabul, Afghanistan; 2020 July.

2. Royo-Cebrecos C, Vilanova D, López J, Arroyo V, Pons M, Francisco G, Carrasco MG, Piqué JM, Sanz S, Dobaño C, García-Basteiro AL. Mass SARS-CoV-2 serological screening, a population-based study in the Principality of Andorra. Lancet Reg Health Eur. 2021 May 21;5:100119. doi: 10.1016/j.lanepe.2021.100119. PMID: 34557824; PMCID: PMC8454851.

3. Tang X, Sharma A, Pasic M, Brown P, Colwill K, Gelband H, Birnboim HC, Nagelkerke N, Bogoch II, Bansal A, Newcombe L, Slater J, Rodriguez PS, Huang G, Fu SH, Meh C, Wu DC, Kaul R, Langlois MA, Morawski E, Hollander A, Eliopoulos D, Aloi B, Lam T, Abe KT, Rathod B, Fazel-Zarandi M, Wang J, Iskilova M, Pasculescu A, Caldwell L, Barrios-Rodiles M, Mohammed-Ali Z, Vas N, Santhanam DR, Cho ER, Qu K, Jha S, Jha V, Suraweera W, Malhotra V, Mastali K, Wen R, Sinha S, Reid A, Gingras AC, Chakraborty P, Slutsky AS, Jha P; Ab-C Study Investigators. Assessment of SARS-CoV-2 Seropositivity During the First and Second Viral Waves in 2020 and 2021 Among Canadian Adults. JAMA Netw Open. 2022 Feb 1;5(2):e2146798. doi: 10.1001/jamanetworkopen.2021.46798. PMID: 35171263; PMCID: PMC8851304.

4. Piler P, Thon V, Andrýsková L, Doležel K, Kostka D, Pavlík T, Dušek L, Pikhart H, Bobák M, Matic S, Klánová J. Nationwide increases in anti-SARS-CoV-2 IgG antibodies between October 2020 and March 2021 in the unvaccinated Czech population. Commun Med (Lond). 2022 Mar 1;2:19. doi: 10.1038/s43856-022-00080-0. PMID: 35603283; PMCID: PMC9053194.

5. Espenhain L, Tribler S, Særke Jørgensen C, Holm Hansen C, Wolff Sönksen U, Ethelberg S. Prevalence of SARS-CoV-2 antibodies in Denmark: nationwide, population-based seroepidemiological study. Eur J Epidemiol. 2021 Jul;36(7):715-725. doi: 10.1007/s10654-021-00796-8. Epub 2021 Aug 22. PMID: 34420152; PMCID: PMC8380416.

6. Ward H, Cooke GS, Atchison C, Whitaker M, Elliott J, Moshe M, Brown JC, Flower B, Daunt A, Ainslie K, Ashby D, Donnelly CA, Riley S, Darzi A, Barclay W, Elliott P. Prevalence of antibody positivity to SARS-CoV-2 following the first peak of infection in England: Serial cross-sectional studies of 365,000 adults. Lancet Reg Health Eur. 2021 May;4:100098. doi: 10.1016/j.lanepe.2021.100098. Epub 2021 May 2. PMID: 33969335; PMCID: PMC8088780.

7. Petersen MS, Strøm M, Fjallsbak JP, Hansen JL, Larsen S, Eliasen EH, Johansen M, Veyhe AS, Kristiansen MF, Weihe P. Low Seroprevalence among Undetected COVID-19 Cases, Faroe Islands, November 2020. Emerg Infect Dis. 2022 Jan;28(1):242-244. doi: 10.3201/eid2801.210917. Epub 2021 Nov 10. PMID: 34757895; PMCID: PMC8714219.

8. Warszawski J, Meyer L, Franck JE, Rahib D, Lydié N, Gosselin A, Counil E, Kreling R, Novelli S, Slama R, Raynaud P, Bagein G, Costemalle V, Sillard P, Fourie T, de Lamballerie X, Bajos N; Epicov Team. Trends in social exposure to SARS-Cov-2 in France. Evidence from the national socio-epidemiological cohort-EPICOV. PLoS One. 2022 May 25;17(5):e0267725. doi: 10.1371/journal.pone.0267725. PMID: 35613100; PMCID: PMC9132278.

9. Carrat F, Lapidus N, Ninove L, Blanché H, Rahib D, Saba Villarroel PM, Touvier M, Severi G, Zins M, Deleuze JF, de Lamballerie X; SAPRIS-SERO study group. Age, COVID-19-like symptoms and SARS-CoV-2 seropositivity profiles after the first wave of the pandemic in France. Infection. 2022 Feb;50(1):257-262. doi: 10.1007/s15010-021-01731-5. Epub 2021 Nov 25. PMID: 34822130; PMCID: PMC8614216.

10. Neuhauser H, Rosario AS, Butschalowsky H, Haller S, Hoebel J, Michel J, Nitsche A, Poethko-Müller C, Prütz F, Schlaud M, Steinhauer HW. Germany's low SARS-CoV-2 seroprevalence confirms effective containment in 2020: Results of the nationwide RKI-SOEP study. medRxiv. 2021 Jan 1.

11. Merkely B, Szabó AJ, Kosztin A, Berényi E, Sebestyén A, Lengyel C, Merkely G, Karády J, Várkonyi I, Papp C, Miseta A, Betlehem J, Burián K, Csóka I, Vásárhelyi B, Ludwig E, Prinz G, Sinkó J, Hankó B, Varga P, Fülöp GÁ, Mag K, Vokó Z; HUNgarian COronaVirus-19 Epidemiological Research (H-UNCOVER) investigators. Novel coronavirus epidemic in the Hungarian population, a cross-sectional nationwide survey to support the exit policy in Hungary. Geroscience. 2020 Aug;42(4):1063-1074. doi: 10.1007/s11357-020-00226-9. Epub 2020 Jul 17. PMID: 32677025; PMCID: PMC7366154.

12. Gudbjartsson DF, Norddahl GL, Melsted P, Gunnarsdottir K, Holm H, Eythorsson E, Arnthorsson AO, Helgason D, Bjarnadottir K, Ingvarsson RF, Thorsteinsdottir B, Kristjansdottir S, Birgisdottir K, Kristinsdottir AM, Sigurdsson MI, Arnadottir GA, Ivarsdottir EV, Andresdottir M, Jonsson F, Agustsdottir AB, Berglund J, Eiriksdottir B, Fridriksdottir R, Gardarsdottir EE, Gottfredsson M, Gretarsdottir OS, Gudmundsdottir S, Gudmundsson KR, Gunnarsdottir TR, Gylfason A, Helgason A, Jensson BO, Jonasdottir A, Jonsson H, Kristjansson T, Kristinsson KG,

Magnusdottir DN, Magnusson OT, Olafsdottir LB, Rognvaldsson S, le Roux L, Sigmundsdottir G, Sigurdsson A, Sveinbjornsson G, Sveinsdottir KE, Sveinsdottir M, Thorarensen EA, Thorbjornsson B, Thordardottir M, Saemundsdottir J, Kristjansson SH, Josefsdottir KS, Masson G, Georgsson G, Kristjansson M, Moller A, Palsson R, Gudnason T, Thorsteinsdottir U, Jonsdottir I, Sulem P, Stefansson K. Humoral Immune Response to SARS-CoV-2 in Iceland. N Engl J Med. 2020 Oct 29;383(18):1724-1734. doi: 10.1056/NEJMoa2026116. Epub 2020 Sep 1. PMID: 32871063; PMCID: PMC7494247.

13. Murhekar MV, Bhatnagar T, Thangaraj JWV, Saravanakumar V, Kumar MS, Selvaraju S, Rade K, Kumar CPG, Sabarinathan R, Turuk A, Asthana S, Balachandar R, Bangar SD, Bansal AK, Chopra V, Das D, Deb AK, Devi KR, Dhikav V, Dwivedi GR, Khan SMS, Kumar MS, Laxmaiah A, Madhukar M, Mahapatra A, Rangaraju C, Turuk J, Yadav R, Andhalkar R, Arunraj K, Bharadwaj DK, Bharti P, Bhattacharya D, Bhat J, Chahal AS, Chakraborty D, Chaudhury A, Deval H, Dhatrak S, Dayal R, Elantamilan D, Giridharan P, Haq I, Hudda RK, Jagjeevan B, Kalliath A, Kanungo S, Krishnan NN, Kshatri JS, Kumar A, Kumar N, Kumar VGV, Lakshmi GGJN, Mehta G, Mishra NK, Mitra A, Nagbhushanam K, Nimmathota A, Nirmala AR, Pandey AK, Prasad GV, Qurieshi MA, Reddy SD, Robinson A, Sahay S, Saxena R, Sekar K, Shukla VK, Singh HB, Singh PK, Singh P, Singh R, Srinivasan N, Varma DS, Viramgami A, Wilson VC, Yadav S, Yadav S, Zaman K, Chakrabarti A, Das A, Dhaliwal RS, Dutta S, Kant R, Khan AM, Narain K, Narasimhaiah S, Padmapriyadarshini C, Pandey K, Pati S, Patil S, Rajkumar H, Ramarao T, Sharma YK, Singh S, Panda S, Reddy DCS, Bhargava B; ICMR Serosurveillance Group. SARS-CoV-2 seroprevalence among the general population and healthcare workers in India, December 2020-January 2021. Int J Infect Dis. 2021 Jul;108:145-155. doi: 10.1016/j.ijid.2021.05.040. Epub 2021 May 19. PMID: 34022338; PMCID: PMC8132496.

14. Khalagi K, Gharibzadeh S, Khalili D, Mansournia MA, Mirab Samiee S, Aghamohamadi S, Mir-Mohammad-Ali Roodaki M, Hashemi SM, Tayeri K, Namdari Tabar H, Azadmanesh K, Tabrizi JS, Mohammad K, Hajipour F, Namaki S, Raeisi A, Ostovar A. Prevalence of COVID-19 in Iran: results of the first survey of the Iranian COVID-19 Serological Surveillance programme. Clin Microbiol Infect. 2021 Nov;27(11):1666-1671. doi: 10.1016/j.cmi.2021.06.002. Epub 2021 Jun 7. PMID: 34111585; PMCID: PMC8226066.

15. Heavey L, Garvey P, Colgan AM, Thornton L, Connell J, Roux T, Hunt M, O'Callaghan F, Culkin F, Keogan M, O'Connor N, O'Sullivan MB, O'Sullivan S, Tait M, De Gascun CF, Igoe D.

The Study to Investigate COVID-19 Infection in People Living in Ireland (SCOPI): A seroprevalence study, June to July 2020. Euro Surveill. 2021 Dec;26(48):2001741. doi: 10.2807/1560-7917.ES.2021.26.48.2001741. PMID: 34857067; PMCID: PMC8641066.
16. Reicher S, Ratzon R, Ben-Sahar S, Hermoni-Alon S, Mossinson D, Shenhar Y, Friger M, Lustig Y, Alroy-Preis S, Anis E, Sadetzki S, Kaliner E. Nationwide seroprevalence of antibodies against SARS-CoV-2 in Israel. Eur J Epidemiol. 2021 Jul;36(7):727-734. doi: 10.1007/s10654-021-00749-1. Epub 2021 Apr 21. PMID: 33884542; PMCID: PMC8059683.
17. Sabbadini LL. Primi risultati dell'indagine di sieroprevalenza SARS-CoV-2. Roma, Istituto Nazionale di Statistica. 2020 Aug 3. Available from: https://www.istat.it/it/files//2020/08/ReportPrimiRisultatiIndagineSiero.pdf.
18. Yoshiyama T, Saito Y, Masuda K, Nakanishi Y, Kido Y, Uchimura K, Mitarai S, Suzuki T, Nakagama Y, Kubota H, Satomi M, Uchikoba S, Ohnishi M, Wakita T, Kato S, Kato K. Prevalence of SARS-CoV-2-Specific Antibodies, Japan, June 2020. Emerg Infect Dis. 2021. Feb;27(2):628-631. doi: 10.3201/eid2702.204088. PMID: 33496235; PMCID: PMC7853542.
19. Government of Jersey. SARS-CoV-2: Prevalence of antibodies in Jersey. St Helier, Statistics Jersey. 2020 May 5. Available from: https://www.gov.je/SiteCollectionDocuments/Government%20and%20administration/R%20Prevalence%20of%20antibodies%202020508%20SJ.pdf.
20. Bellizzi S, Alsawalha L, Sheikh Ali S, Sharkas G, Muthu N, Ghazo M, Hayajneh W, Profili MC, Obeidat NM. A three-phase population based sero-epidemiological study: Assessing the trend in prevalence of SARS-CoV-2 during COVID-19 pandemic in Jordan. One Health. 2021 Jul 10;13:100292. doi: 10.1016/j.onehlt.2021.100292. PMID: 34295958; PMCID: PMC8272624.
21. Virachith S, Pommelet V, Calvez E, Khounvisith V, Sayasone S, Kounnavong S, Maxay M, Xangsayarath P, Temmam S, Eloit M, Escriou N, Rose T, Vongphayloth K, Hübschen JM, Lacoste V, Somlor S, Phonekeo D, Brey PT, Black AP. Low seroprevalence of COVID-19 in Lao PDR, late 2020. Lancet Reg Health West Pac. 2021 Aug;13:100197. doi: 10.1016/j.lanwpc.2021.100197. Epub 2021 Jul 14. PMID: 34278365; PMCID: PMC8277598.
22. Hoballah A, El Haidari R, Siblany G, Abdel Sater F, Mansour S, Hassan H, Abou-Abbas L. SARS-CoV-2 antibody seroprevalence in Lebanon: findings from the first nationwide serosurvey. BMC Infect Dis. 2022 Jan 10;22(1):42. doi: 10.1186/s12879-022-07031-z. PMID: 35012464; PMCID: PMC8744021.

23. Šmigelskas K, Petrikonis K, Kasiulevičius V, Kalėdienė R, Jakaitienė A, Kaselienė S, Sauliūnė S, Beržanskytė A, Stankūnas M. SARS-CoV-2 Seroprevalence in Lithuania: Results of National Population Survey. Acta Med Litu. 2021;28(1):48-58. doi: 10.15388/Amed.2020.28.1.2. Epub 2021 Jan 18. PMID: 34393628; PMCID: PMC8311832.

24. Abdul-Raheem R, Moosa S, Waheed F, Aboobakuru M, Ahmed IN, Rafeeg FN, Saeed M. A sero-epidemiological study after two waves of the COVID-19 epidemic. Asian Pac J Allergy Immunol. 2021 Dec 26. doi: 10.12932/AP-040721-1177. Epub ahead of print. PMID: 34953474.

25. Basto-Abreu A, Carnalla M, Torres-Ibarra L, Romero-Martínez M, Martínez-Barnetche J, López-Martínez I, Aparicio-Antonio R, Shamah-Levy T, Alpuche-Aranda C, Rivera JA, Barrientos-Gutierrez T; ENSANUT-COVID collaborators. Nationally representative SARS-CoV-2 antibody prevalence estimates after the first epidemic wave in Mexico. Nat Commun. 2022 Feb 1;13(1):589. doi: 10.1038/s41467-022-28232-9. PMID: 35105873; PMCID: PMC8807586.

26. Chimeddorj B, Mandakh U, Le LV, Bayartsogt B, Deleg Z, Enebish O, Altanbayar O, Magvan B, Gantumur A, Byambaa O, Enebish G, Saindoo BE, Davaadorj M, Amgalanbaatar A, Enkhtugs K, Munkhbayar U, Bayanjargal B, Badamsambuu T, Dashtseren M, Narmandakh Z, Togoo K, Boldbaatar EA, Bat-Erdene A, Mukhtar Y, Shagdarsuren OE, Ganbat M, Batjargal O, Bavuusuren B, Batchuluun B, Zulmunkh G, Byambatsogt G, Nyamdavaa K, Dalkh T, Boldbaatar D, Tseren T, Gantulga D, Damdinbazar O, Vanchin B, Subissi L, Bergeri I, Dambadarjaa D, Pagbajabyn N, Greif G, Erkhembayar R. SARS-CoV-2 seroprevalence in Mongolia: Results from a national population survey. Lancet Reg Health West Pac. 2021 Dec;17:100317. doi: 10.1016/j.lanwpc.2021.100317. Epub 2021 Nov 23. PMID: 34841381; PMCID: PMC8609908.

27. Government of Nepal. Enhanced surveillance on sero-prevalence of SARS-CoV-2 in general population. Kathmandu, Government of Nepal Ministry of Health and Population. 2021 Apr 4. Available from: https://mohp.gov.np/attachments/article/708/First%20Sero-prevalence_final_report_04-04-2021.pdf.

28. Vos ERA, van Boven M, den Hartog G, Backer JA, Klinkenberg D, van Hagen CCE, Boshuizen H, van Binnendijk RS, Mollema L, van der Klis FRM, de Melker HE. Associations Between Measures of Social Distancing and Severe Acute Respiratory Syndrome Coronavirus 2 Seropositivity: A Nationwide Population-based Study in the Netherlands. Clin Infect Dis. 2021 Dec 16;73(12):2318-2321. doi: 10.1093/cid/ciab264. PMID: 33772265; PMCID: PMC8083720.

29. Anda EE, Braaten T, Borch KB, Nøst TH, Chen SLF, Lukic M, Lund E, Forland F, Leon DA, Winje BA, Kran AB, Kalager M, Johansen FL, Sandanger TM. Seroprevalence of antibodies against SARS-CoV-2 in the adult population during the pre-vaccination period, Norway, winter 2020/21. Euro Surveill. 2022 Mar;27(13):2100376. doi: 10.2807/1560-7917.ES.2022.27.13.2100376. PMID: 35362405; PMCID: PMC8973017.
30. Al-Abri SS, Al-Wahaibi A, Al-Kindi H, Kurup PJ, Al-Maqbali A, Al-Mayahi Z, Al-Tobi MH, Al-Katheri SH, Albusaidi S, Al-Sukaiti MH, Al Balushi AYM, Abdelgadir IO, Al-Shehi N, Morkos E, Al-Maani A, Al-Rawahi B, Alyaquobi F, Alqayoudhi A, Al-Harthy K, Al-Khalili S, Al-Rashdi A, Al-Shukri I, Al Ghafri TS, Al-Hashmi F, Al Jassasi SM, Alshaqsi N, Mitra N, Al Aamry HS, Shah P, Al Marbouai HH, Al Araimi AH, Kair IM, Al Manji AM, Almallak AS, Al Alawi FK, Vaidya V, Muqeetullah M, Alrashdi H, Al Jamoudi SSN, Alshaqsi A, Al Sharji A, Al Shukeiri H, Al-Abri B, Al-Rawahi S, Al-Lamki SH, Al-Manji A, Al-Jardani A. Seroprevalence of SARS-CoV-2 antibodies in the general population of Oman: results from four successive nationwide sero-epidemiological surveys. Int J Infect Dis. 2021 Nov;112:269-277. doi: 10.1016/j.ijid.2021.09.062. Epub 2021 Sep 30. PMID: 34601146; PMCID: PMC8482550.
31. Ahmad AM, Shahzad K, Masood M, Umar M, Abbasi F, Hafeez A. COVID-19 seroprevalence in Pakistan: a cross-sectional study. BMJ Open. 2022 Apr 6;12(4):e055381. doi: 10.1136/bmjopen-2021-055381. PMID: 35387815; PMCID: PMC8987211.
32. Canto E Castro L, Gomes A, Serrano M, Pereira AHG, Ribeiro R, Napoleão P, Domingues I, Silva C, Fanczal J, Afonso Â, Lopes A, Toader I, de Sousa MJR, de Sousa JGR, de Sousa G, Mota MM, Silva-Santos B, Veldhoen M, Ribeiro RM. Longitudinal SARS-CoV-2 seroprevalence in Portugal and antibody maintenance 12 months after infection. Eur J Immunol. 2022 Jan;52(1):149-160. doi: 10.1002/eji.202149619. Epub 2021 Nov 10. PMID: 34695227; PMCID: PMC8646574.
33. Popova AY, Smirnov VS, Andreeva EE, Babura EA, Balakhonov SV, Bashketova NS, Bugorkova SA, Bulanov MV, Valeullina NN, Vetrov VV, Goryaev DV, Detkovskaya TN, Ezhlova EB, Zaitseva NN, Istorik OA, Kovalchuk IV, Kozlovskikh DN, Kombarova SY, Kurganova OP, Lomovtsev AE, Lukicheva LA, Lyalina LV, Melnikova AA, Mikailova OM, Noskov AK, Noskova LN, Oglezneva EE, Osmolovskaya TP, Patyashina MA, Penkovskaya NA, Samoilova LV, Stepanova TF, Trotsenko OE, Totolian AA. SARS-CoV-2 Seroprevalence Structure of the Russian Population during the COVID-19 Pandemic. Viruses. 2021 Aug 19;13(8):1648. doi: 10.3390/v13081648. PMID: 34452512; PMCID: PMC8402751.

34. Talla C, Loucoubar C, Roka JL, Barry MA, Ndiaye S, Diarra M, Thiam MS, Faye O, Dia M, Diop M, Ndiaye O. Seroprevalence of anti-SARS-CoV-2 antibodies in Senegal: a national population-based cross-sectional survey, between October and November 2020. IJID Regions. 2022 Jun 1;3:117-25.

35. Poljak M, Oštrbenk Valenčak A, Štrumbelj E, Maver Vodičar P, Vehovar V, Resman Rus K, Korva M, Knap N, Seme K, Petrovec M, Zupan B, Demšar J, Kurdija S, Avšič Županc T. Seroprevalence of severe acute respiratory syndrome coronavirus 2 in Slovenia: results of two rounds of a nationwide population study on a probability-based sample, challenges and lessons learned. Clin Microbiol Infect. 2021 Jul;27(7):1039.e1-1039.e7. doi: 10.1016/j.cmi.2021.03.009. Epub 2021 Apr 7. PMID: 33838303; PMCID: PMC8064903.

36. Gobierno de España. Estudio ENE-COVID: Cuarta ronda: Estudio nacional de sero-epidemiología de la infección por SARS-CoV-2 en España. Madrid, Gobierno de España Ministerio de Ciencia y Innovación. 2020 Dec 15. Available from: https://www.sanidad.gob.es/gabinetePrensa/notaPrensa/pdf/15.12151220163348113.pdf

37. Sullivan PS, Siegler AJ, Shioda K, Hall EW, Bradley H, Sanchez T, Luisi N, Valentine-Graves M, Nelson KN, Fahimi M, Kamali A, Sailey C, Lopman BA. Severe Acute Respiratory Syndrome Coronavirus 2 Cumulative Incidence, United States, August 2020-December 2020. Clin Infect Dis. 2022 Apr 9;74(7):1141-1150. doi: 10.1093/cid/ciab626. PMID: 34245245; PMCID: PMC8406864.

38. Kalish H, Klumpp-Thomas C, Hunsberger S, Baus HA, Fay MP, Siripong N, Wang J, Hicks J, Mehalko J, Travers J, Drew M, Pauly K, Spathies J, Ngo T, Adusei KM, Karkanitsa M, Croker JA, Li Y, Graubard BI, Czajkowski L, Belliveau O, Chairez C, Snead KR, Frank P, Shunmugavel A, Han A, Giurgea LT, Rosas LA, Bean R, Athota R, Cervantes-Medina A, Gouzoulis M, Heffelfinger B, Valenti S, Caldararo R, Kolberg MM, Kelly A, Simon R, Shafiq S, Wall V, Reed S, Ford EW, Lokwani R, Denson JP, Messing S, Michael SG, Gillette W, Kimberly RP, Reis SE, Hall MD, Esposito D, Memoli MJ, Sadtler K. Undiagnosed SARS-CoV-2 seropositivity during the first 6 months of the COVID-19 pandemic in the United States. Sci Transl Med. 2021 Jul 7;13(601):eabh3826. doi: 10.1126/scitranslmed.abh3826. Epub 2021 Jun 22. PMID: 34158410; PMCID: PMC8432952.

Appendix. PRISMA 2020 checklists (Main article and Abstract)

Section and Topic	Item #	Checklist item	Location where item is reported (page)
**TITLE**			
Title	1	Identify the report as a systematic review.	1
**ABSTRACT**			
Abstract	2	See the PRISMA 2020 for Abstracts checklist.	3
**INTRODUCTION**			
Rationale	3	Describe the rationale for the review in the context of existing knowledge.	5
Objectives	4	Provide an explicit statement of the objective(s) or question(s) the review addresses.	5,6
**METHODS**			
Eligibility criteria	5	Specify the inclusion and exclusion criteria for the review and how studies were grouped for the syntheses.	6
Information sources	6	Specify all databases, registers, websites, organisations, reference lists and other sources searched or consulted to identify studies. Specify the date when each source was last searched or consulted.	6
Search strategy	7	Present the full search strategies for all databases, registers and websites, including any filters and limits used.	6
Selection process	8	Specify the methods used to decide whether a study met the inclusion criteria of the review, including how many reviewers screened each record and each report retrieved, whether they worked independently, and if applicable, details of automation tools used in the process.	6,7
Data collection process	9	Specify the methods used to collect data from reports, including how many reviewers collected data from each report, whether they worked independently, any processes for obtaining or confirming data from study investigators, and if applicable, details of automation tools used in the process.	7
Data items	10a	List and define all outcomes for which data were sought. Specify whether all results that were compatible with each outcome domain in each study were sought (e.g. for all measures, time points, analyses), and if not, the methods used to decide which results to collect.	7,8
	10b	List and define all other variables for which data were sought (e.g. participant and intervention characteristics, funding sources). Describe any assumptions made about any missing or unclear information.	7,8
Study risk of bias assessment	11	Specify the methods used to assess risk of bias in the included studies, including details of the tool(s) used, how many reviewers assessed each study and whether they worked independently, and if applicable, details of automation tools used in the process.	6
Effect measures	12	Specify for each outcome the effect measure(s) (e.g. risk ratio, mean difference) used in the synthesis or presentation of results.	8
Synthesis methods	13a	Describe the processes used to decide which studies were eligible for each synthesis (e.g. tabulating the study intervention characteristics and comparing against the planned groups for each synthesis (item #5)).	8,9
	13b	Describe any methods required to prepare the data for presentation or synthesis, such as handling of missing summary statistics, or data conversions.	8,9
	13c	Describe any methods used to tabulate or visually display results of individual studies and syntheses.	8,9
	13d	Describe any methods used to synthesize results and provide a rationale for the choice(s). If meta-analysis was performed, describe the model(s), method(s) to identify the presence and extent of statistical heterogeneity, and software package(s) used.	8,9
	13e	Describe any methods used to explore possible causes of heterogeneity among study results (e.g. subgroup analysis, meta-regression).	9
	13f	Describe any sensitivity analyses conducted to assess robustness of the synthesized results.	9

Section and Topic	Item #	Checklist item	Location where item is reported (page)
Reporting bias assessment	14	Describe any methods used to assess risk of bias due to missing results in a synthesis (arising from reporting biases).	-
Certainty assessment	15	Describe any methods used to assess certainty (or confidence) in the body of evidence for an outcome.	-
**RESULTS**			
Study selection	16a	Describe the results of the search and selection process, from the number of records identified in the search to the number of studies included in the review, ideally using a flow diagram.	9,27,30,31
	16b	Cite studies that might appear to meet the inclusion criteria, but which were excluded, and explain why they were excluded.	30,31
Study characteristics	17	Cite each included study and present its characteristics.	38-44
Risk of bias in studies	18	Present assessments of risk of bias for each included study.	9
Results of individual studies	19	For all outcomes, present, for each study: (a) summary statistics for each group (where appropriate) and (b) an effect estimate and its precision (e.g. confidence/credible interval), ideally using structured tables or plots.	9,24,28,29
Results of syntheses	20a	For each synthesis, briefly summarise the characteristics and risk of bias among contributing studies.	-
	20b	Present results of all statistical syntheses conducted. If meta-analysis was done, present for each the summary estimate and its precision (e.g. confidence/credible interval) and measures of statistical heterogeneity. If comparing groups, describe the direction of the effect.	10,11
	20c	Present results of all investigations of possible causes of heterogeneity among study results.	11,12
	20d	Present results of all sensitivity analyses conducted to assess the robustness of the synthesized results.	11
Reporting biases	21	Present assessments of risk of bias due to missing results (arising from reporting biases) for each synthesis assessed.	-
Certainty of evidence	22	Present assessments of certainty (or confidence) in the body of evidence for each outcome assessed.	-
**DISCUSSION**			
Discussion	23a	Provide a general interpretation of the results in the context of other evidence.	12
	23b	Discuss any limitations of the evidence included in the review.	12-15
	23c	Discuss any limitations of the review processes used.	13
	23d	Discuss implications of the results for practice, policy, and future research.	15
**OTHER INFORMATION**			
Registration and protocol	24a	Provide registration information for the review, including register name and registration number, or state that the review was not registered.	6
	24b	Indicate where the review protocol can be accessed, or state that a protocol was not prepared.	6
	24c	Describe and explain any amendments to information provided at registration or in the protocol.	29
Support	25	Describe sources of financial or non-financial support for the review, and the role of the funders or sponsors in the review.	1,2
Competing	26	Declare any competing interests of review authors.	2

Section and Topic	Item #	Checklist item	Location where item is reported (page)
interests			
Availability of data, code and other materials	27	Report which of the following are publicly available and where they can be found: template data collection forms; data extracted from included studies; data used for all analyses; analytic code; any other materials used in the review.	-

From: Page MJ, McKenzie JE, Bossuyt PM, Boutron I, Hoffmann TC, Mulrow CD, et al. The PRISMA 2020 statement: an updated guideline for reporting systematic reviews. BMJ 2021;372:n71. doi: 10.1136/bmj.n71

For more information, visit: http://www.prisma-statement.org/

Section and Topic	Item #	Checklist item	Reported (Yes/No)
**TITLE**			
Title	1	Identify the report as a systematic review.	Yes
**BACKGROUND**			
Objectives	2	Provide an explicit statement of the main objective(s) or question(s) the review addresses.	Yes
**METHODS**			
Eligibility criteria	3	Specify the inclusion and exclusion criteria for the review.	Yes
Information sources	4	Specify the information sources (e.g. databases, registers) used to identify studies and the date when each was last searched.	Yes
Risk of bias	5	Specify the methods used to assess risk of bias in the included studies.	Yes
Synthesis of results	6	Specify the methods used to present and synthesise results.	Yes
**RESULTS**			
Included studies	7	Give the total number of included studies and participants and summarise relevant characteristics of studies.	Yes
Synthesis of results	8	Present results for main outcomes, preferably indicating the number of included studies and participants for each. If meta-analysis was done, report the summary estimate and confidence/credible interval. If comparing groups, indicate the direction of the effect (i.e. which group is favoured).	Yes
**DISCUSSION**			
Limitations of evidence	9	Provide a brief summary of the limitations of the evidence included in the review (e.g. study risk of bias, inconsistency and imprecision).	Yes
Interpretation	10	Provide a general interpretation of the results and important implications.	Yes
**OTHER**			
Funding	11	Specify the primary source of funding for the review.	Yes

Section and Topic	Item #	Checklist item	Reported (Yes/No)
Registration	12	Provide the register name and registration number.	Yes

From: Page MJ, McKenzie JE, Bossuyt PM, Boutron I, Hoffmann TC, Mulrow CD, et al. The PRISMA 2020 statement: an updated guideline for reporting systematic reviews. BMJ 2021;372:n71. doi: 10.1136/bmj.n71

For more information, visit: http://www.prisma-statement.org/
